# Supplementary material for: Escape from X inactivation varies across genes and tissues and shapes sex-biased sex chromosome gene expression
Source: BMC Genomics. 2026 Feb 19;27:304. doi: 10.1186/s12864-026-12611-3 (PMC13020151; doi:10.1186/s12864-026-12611-3)
Supplement: Supplementary file 1 — Supplementary Material 1. [file 12864_2026_12611_MOESM1_ESM.pdf]

Supplementary Figures

Figure S1 | Sex effects are consistent across datasets

Sex-biased expression (β_{mash}) from DeCasien et al. 2025 (y-axis) versus sex-biased expression (β_{mash}) from Oliva et al. 2020 (x-axis) for overlapping genes in each tissue (see legend) (the latter only published the top 500 significant genes per tissue).

Figure S2 | Mean expression level is not consistently linked to XCI escape or sex-bias

Distributions, scatterplots, and Spearman's rank order correlation results for pairwise comparisons of allelic expression, sex-biased expression, and mean expression level (see Methods). Colors represent gene sets included in Figure 1D (red = female-biased NPX escape genes; green = female-biased NPX inactive/silenced genes; blue = male-biased PAR escape genes; purple = male-biased PAR inactive/silenced genes). *$0.01<p<0.05$. **$0.001<p<0.01$. ***$p<0.001$.

Figure S3 | Female-biased expression is not a perfect proxy for XCI escape

A) Bar charts showing the number (top) or proportion (bottom) of gene x tissue observations within different categories of XCI escape (see legend) within each gene-level category (x-axis) (Table S1).

B) Tile plot showing gene x tissue-level data from Figure S3A.

C) Bar charts showing the number (top) or proportion (bottom) of PAR gene x tissue observations showing sex-biased expression (see legend) within each tissue-level XCI category (x-axis) (Table S5). Reproduced in Figure 1A.

D) Tile plot showing gene x tissue-level data from Figures S3C and E.

E) Bar charts showing the number (top) or proportion (bottom) of NPX gene x tissue observations showing sex-biased expression (see legend) within each tissue-level XCI category (x-axis) (Table S5). Reproduced in Figure 1A.

Figure S4 | Single cell datasets recover cross-TAD variation in PAR gene escape from XCI

A) Boxplots of XCI escape (X_i / total expression) for PAR genes from single cell data, separated by TADs (see Figure 1C). Data is from 4 datasets (see Figure S4B) and values were averaged across samples (within cell types and datasets). Mean values across observations within each TAD are shown in red. ANOVA results are shown above the plot. Pairwise significant differences (Tukey HSD $p_{adj}<0.05$) are indicated with brackets.

B) Boxplots of XCI escape (X_i / total expression) for PAR genes from 4 single cell datasets, separated by TADs (see Figure 1A). Values were averaged across samples (within cell types and datasets). These data are summarized across datasets in Figure S4A. Mean values across observations within each TAD are shown in red.

C) Boxplots of XCI escape (X_i / total expression) for PAR genes from single cell data, separated by TADs (see Figure 1A). Data is from 2 datasets (see Figure S4D) for which data was available per sample. Mean values across observations within each TAD are shown in red. ANOVA results are shown above the plot. Pairwise significant differences (Tukey HSD $p_{adj}<0.05$) are indicated with brackets.

D) Boxplots of XCI escape (X_i / total expression) for PAR genes from 2 single cell datasets (per sample data), separated by TADs. These data are summarized across datasets in Figure S4C. Mean values across observations within each TAD are shown in red.

Figure S5 | Relationships between XCI escape and sex-biased expression vary across genes and tissues

A) Similar to Figure 1D, but for all genes (Table S5).

B) Similar to Figure 1D, but for individual tissues (Table S6).

C) Sex-biased expression (β_{mash}) (x-axis) for PAR gene x tissues that show nearly full escape from XCI (i.e., nearly equal expression from X_i and X_a; 1-AE>0.99). Shapes indicate tissue (see legend). Color indicates sex-bias (see legend).

D) Similar to Figure 1D, but for individual genes (Table S7). Shapes indicate tissue (see legend). Partially reproduced in Figure 1E.

Figure S6 | Genes that escape XCI and exhibit sex-biased expression also show sex-biased enhancer activity

Count of sex-biased peaks (Table S4) (female- or male-biased; bottom) near genes in each sex-biased expression (male- or female-biased; left) category and gene set (NPX vs PAR; top).

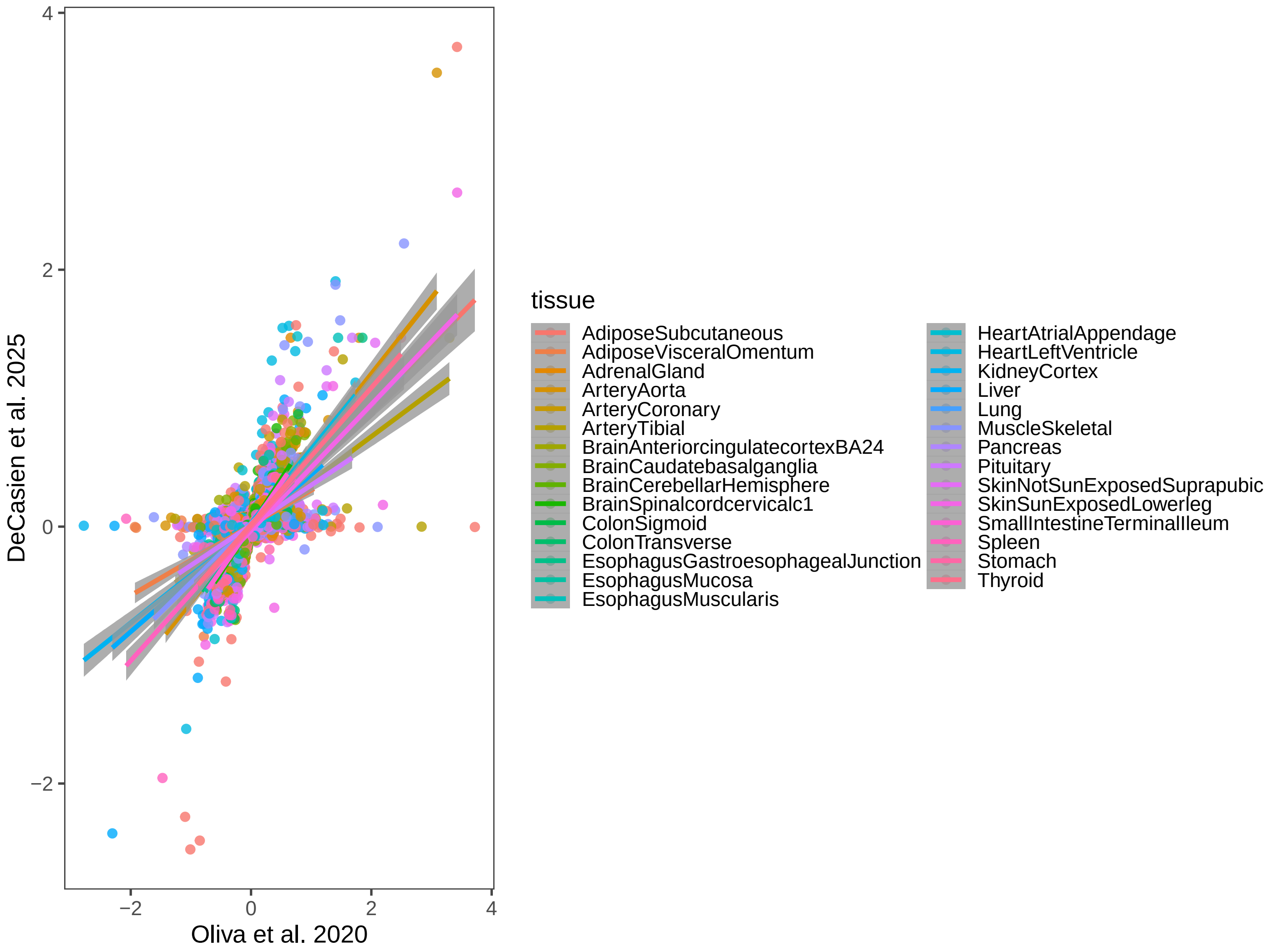

DeCasien et al. 2025
Oliva et al. 2020
4
2
0
−2
−2
0
2
4
tissue
AdiposeSubcutaneous
AdiposeVisceralOmentum
AdrenalGland
ArteryAorta
ArteryCoronary
ArteryTibial
BrainAnteriorcingulatecortexBA24
BrainCaudatebasalganglia
BrainCerebellarHemisphere
BrainSpinalcordcervicalc1
ColonSigmoid
ColonTransverse
EsophagusGastroesophagealJunction
EsophagusMucosa
EsophagusMuscularis
HeartAtrialAppendage
HeartLeftVentricle
KidneyCortex
Liver
Lung
MuscleSkeletal
Pancreas
Pituitary
SkinNotSunExposedSuprapubic
SkinSunExposedLowerleg
SmallIntestineTerminalIleum
Spleen
Stomach
Thyroid

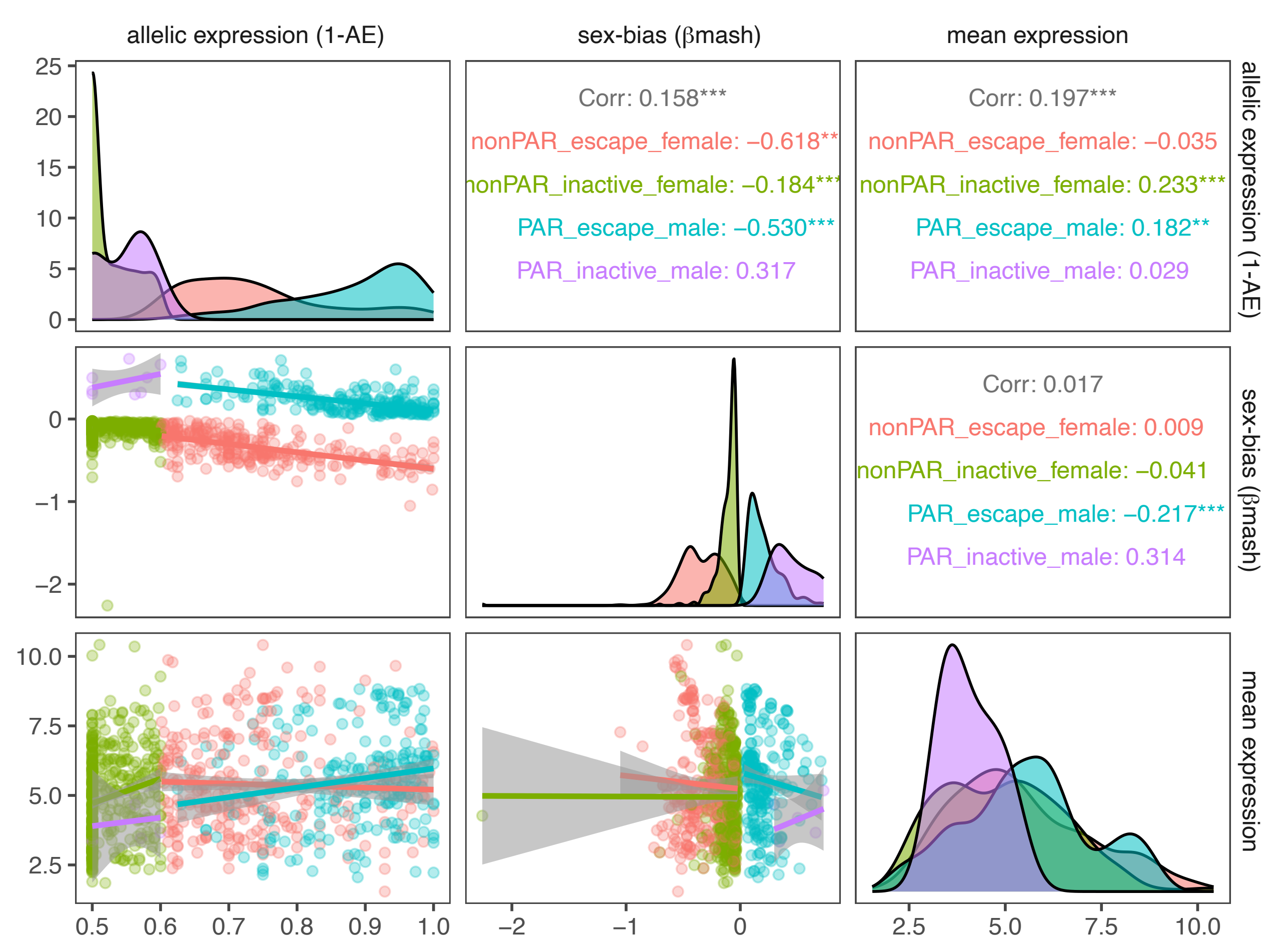
allelic expression (1-AE)
sex-bias (βmash)
mean expression
Corr: 0.158***
nonPAR_escape_female: –0.618**
nonPAR_inactive_female: –0.184***
PAR_escape_male: –0.530***
PAR_inactive_male: 0.317
Corr: 0.197***
nonPAR_escape_female: –0.035
nonPAR_inactive_female: 0.233***
PAR_escape_male: 0.182**
PAR_inactive_male: 0.029
Corr: 0.017
nonPAR_escape_female: 0.009
nonPAR_inactive_female: –0.041
PAR_escape_male: –0.217***
PAR_inactive_male: 0.314
allelic expression (1-AE)
sex-bias (βmash)
mean expression

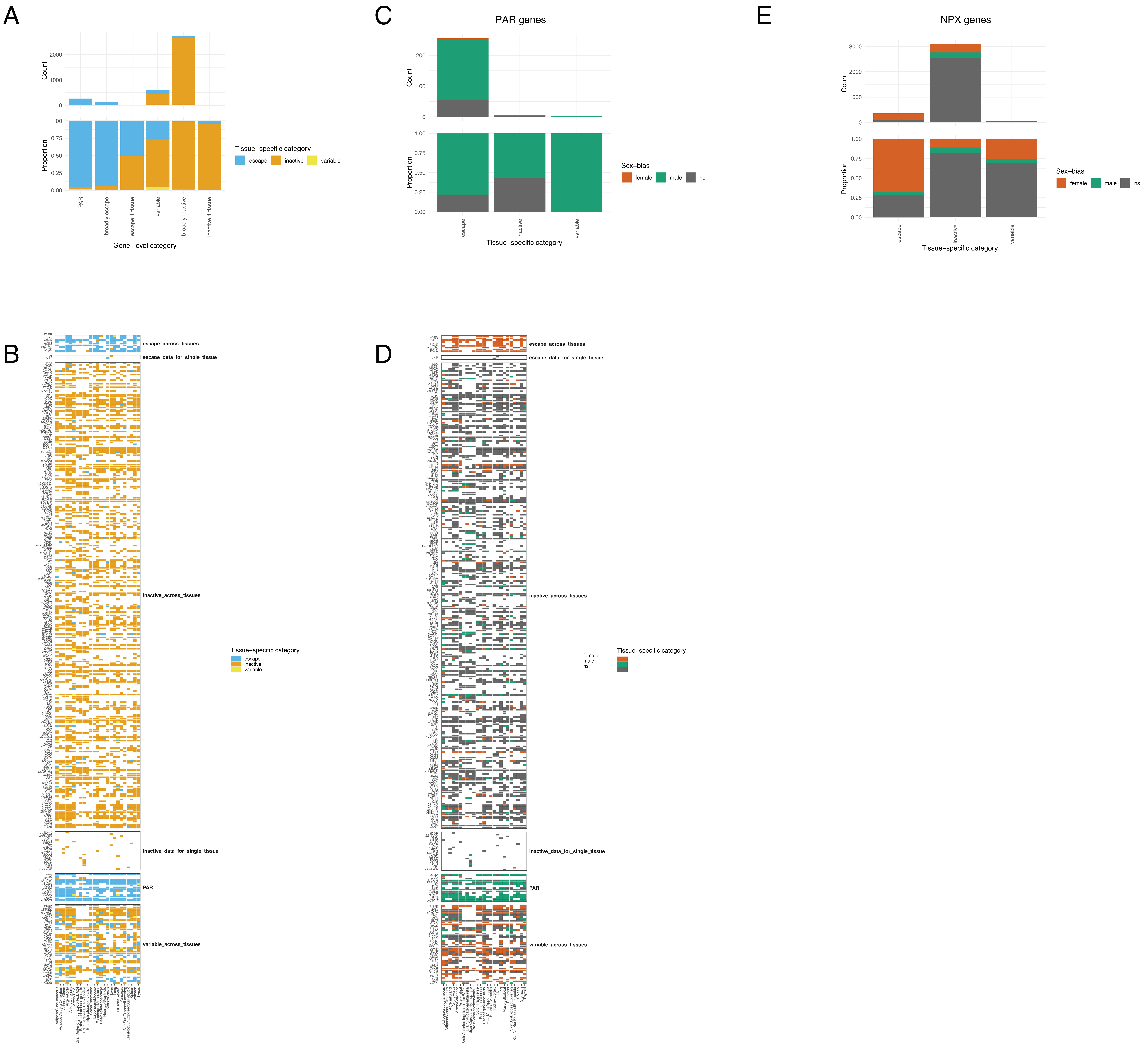

A
Count
2000
1000
0
Proportion
1.00
0.75
0.50
0.25
0.00
PAR
broadly escape
escape 1 tissue
variable
broadly inactive
inactive 1 tissue
Gene−level category
Tissue−specific category
escape
inactive
variable
C
PAR genes
Count
200
100
0
Proportion
1.00
0.75
0.50
0.25
0.00
escape
inactive
variable
Tissue−specific category
Sex−bias
female
male
ns
E
NPX genes
Count
3000
2000
1000
0
Proportion
1.00
0.75
0.50
0.25
0.00
escape
inactive
variable
Tissue−specific category
Sex−bias
female
male
ns
B
escape_across_tissues
escape_data_for_single_tissue
inactive_across_tissues
inactive_data_for_single_tissue
PAR
variable_across_tissues
Tissue−specific category
escape
inactive
variable
D
escape_across_tissues
escape_data_for_single_tissue
inactive_across_tissues
inactive_data_for_single_tissue
PAR
variable_across_tissues
female
male
ns
Tissue−specific category

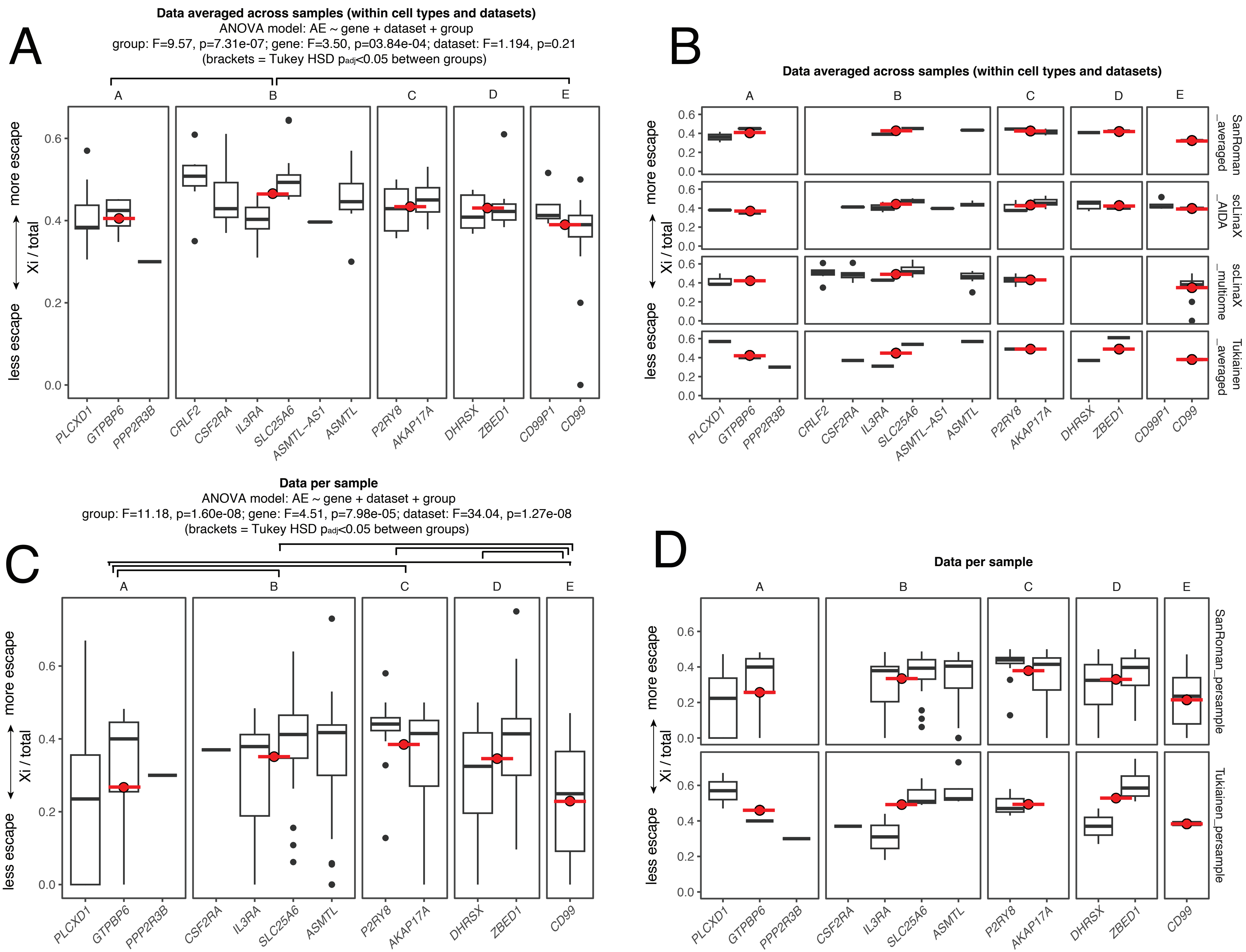
A
Data averaged across samples (within cell types and datasets)
ANOVA model: AE ~ gene + dataset + group
group: F=9.57, p=7.31e-07; gene: F=3.50, p=03.84e-04; dataset: F=1.194, p=0.21
(brackets = Tukey HSD padj<0.05 between groups)
more escape
less escape
Xi / total
PLCXD1
GTPBP6
PPP2R3B
CRLF2
CSF2RA
IL3RA
SLC25A6
ASMTL-AS1
ASMTL
P2RY8
AKAP17A
DHRSX
ZBED1
CD99P1
CD99
B
Data averaged across samples (within cell types and datasets)
SanRoman_averaged
scLinaX_AIDA
scLinaX_multiome
Tukiainen_averaged
C
Data per sample
ANOVA model: AE ~ gene + dataset + group
group: F=11.18, p=1.60e-08; gene: F=4.51, p=7.98e-05; dataset: F=34.04, p=1.27e-08
(brackets = Tukey HSD padj<0.05 between groups)
D
Data per sample
SanRoman_persample
Tukiainen_persample

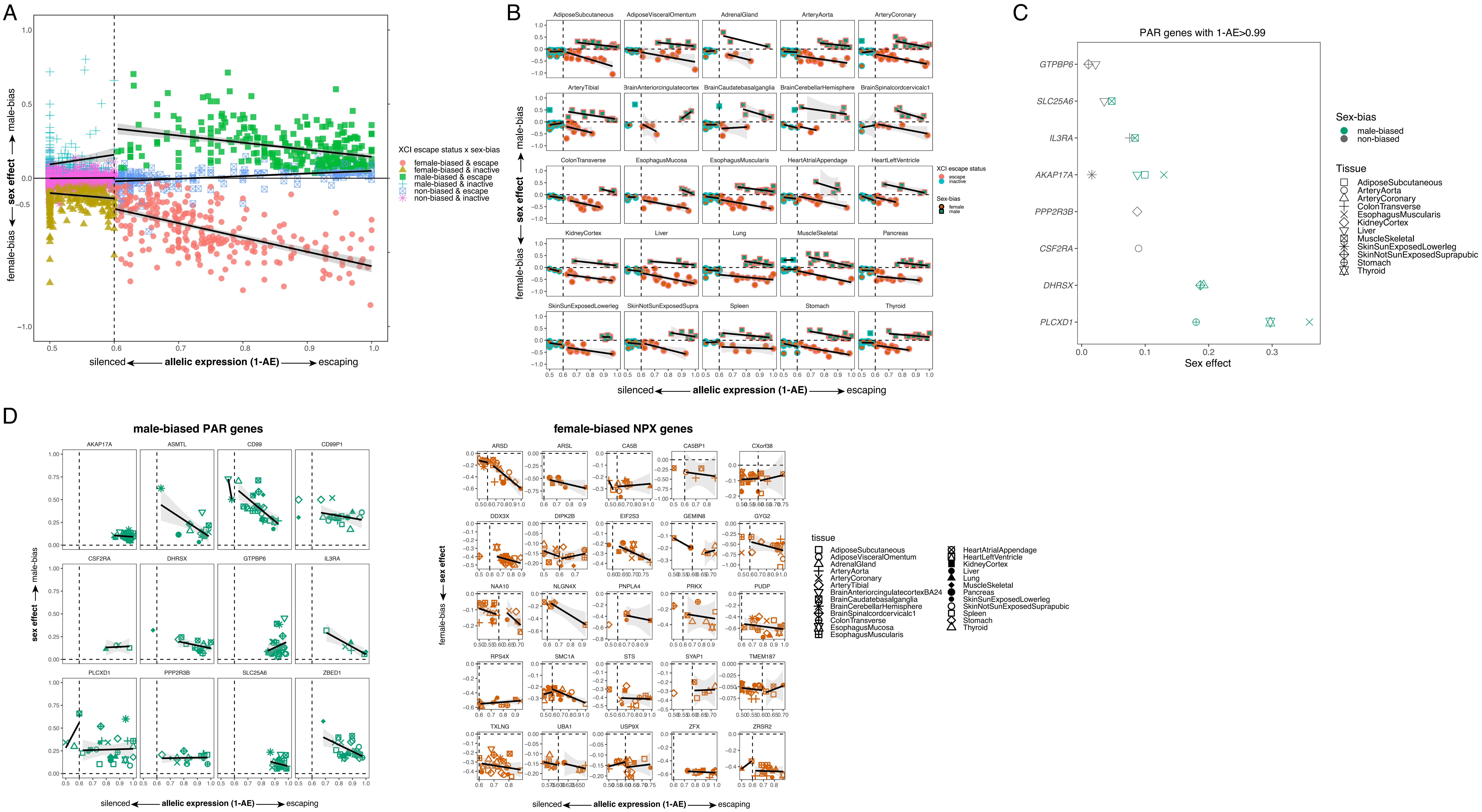
A
male-bias
sex effect
female-bias
silenced
allelic expression (1-AE)
escaping
XCI escape status x sex-bias
female-biased & escape
female-biased & inactive
male-biased & escape
male-biased & inactive
non-biased & escape
non-biased & inactive
B
AdiposeSubcutaneous
AdiposeVisceralOmentum
AdrenalGland
ArteryAorta
ArteryCoronary
ArteryTibial
BrainAnteriorcingulatecortex
BrainCaudatebasalganglia
BrainCerebellarHemisphere
BrainSpinalcordcervicalc1
ColonTransverse
EsophagusMucosa
EsophagusMuscularis
HeartAtrialAppendage
HeartLeftVentricle
KidneyCortex
Liver
Lung
MuscleSkeletal
Pancreas
SkinSunExposedLowerleg
SkinNotSunExposedSupra
Spleen
Stomach
Thyroid
XCI escape status
escape
inactive
Sex-bias
female
male
C
PAR genes with 1-AE>0.99
GTPBP6
SLC25A6
IL3RA
AKAP17A
PPP2R3B
CSF2RA
DHRSX
PLCXD1
Sex effect
Sex-bias
male-biased
non-biased
Tissue
AdiposeSubcutaneous
ArteryAorta
ArteryCoronary
ColonTransverse
EsophagusMuscularis
KidneyCortex
Liver
MuscleSkeletal
SkinSunExposedLowerleg
SkinNotSunExposedSuprapubic
Stomach
Thyroid
D
male-biased PAR genes
AKAP17A
ASMTL
CD99
CD99P1
CSF2RA
DHRSX
GTPBP6
IL3RA
PLCXD1
PPP2R3B
SLC25A6
ZBED1
female-biased NPX genes
ARSD
ARSL
CA5B
CA5BP1
CXorf38
DDX3X
DIPK2B
EIF2S3
GEMIN8
GYG2
NAA10
NLGN4X
PNPLA4
PRKX
PUDP
RPS4X
SMC1A
STS
SYAP1
TMEM187
TXLNG
UBA1
USP9X
ZFX
ZRSR2
tissue
AdiposeSubcutaneous
AdiposeVisceralOmentum
AdrenalGland
ArteryAorta
ArteryCoronary
ArteryTibial
BrainAnteriorcingulatecortexBA24
BrainCaudatebasalganglia
BrainCerebellarHemisphere
BrainSpinalcordcervicalc1
ColonTransverse
EsophagusMucosa
EsophagusMuscularis
HeartAtrialAppendage
HeartLeftVentricle
KidneyCortex
Liver
Lung
MuscleSkeletal
Pancreas
SkinSunExposedLowerleg
SkinNotSunExposedSuprapubic
Spleen
Stomach
Thyroid

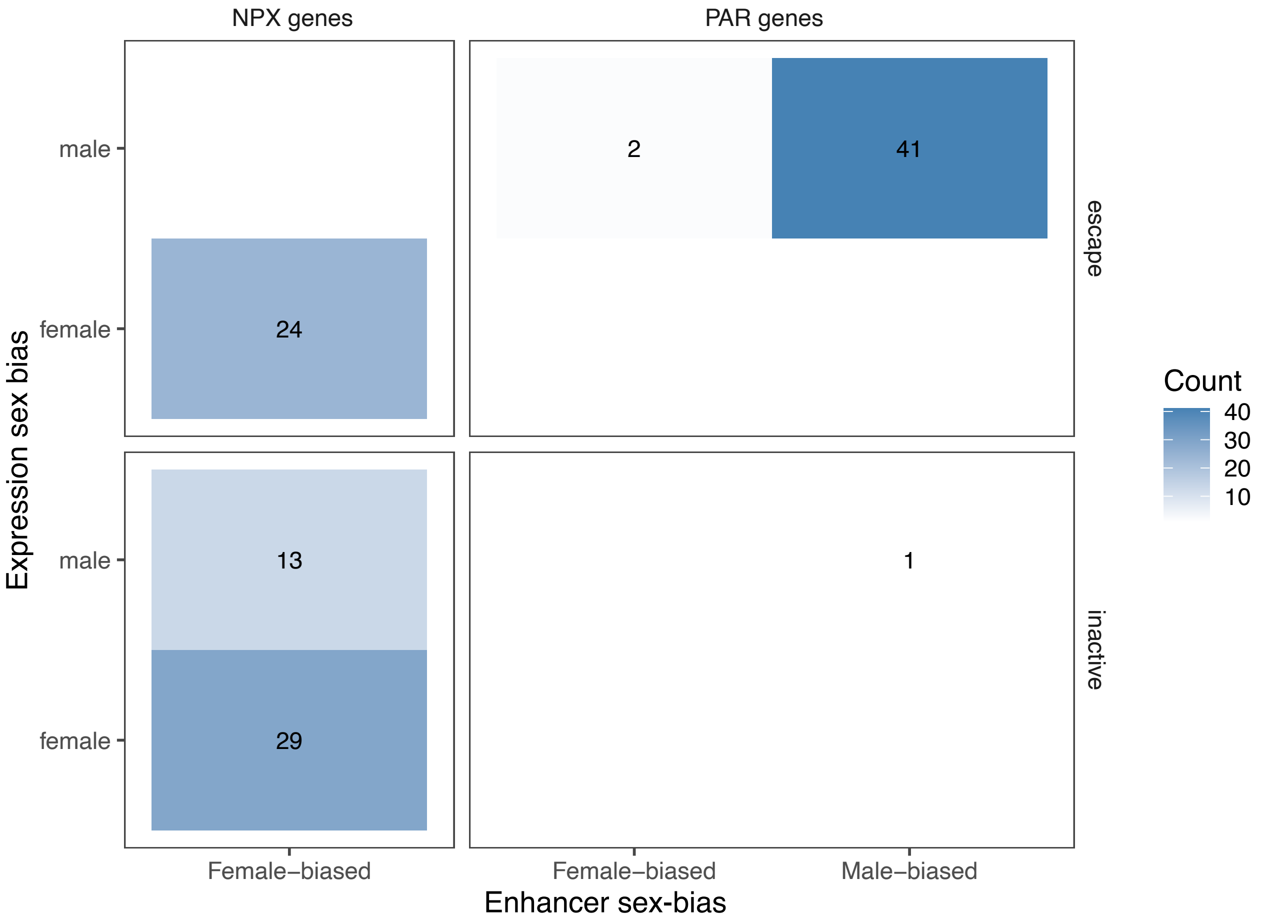
NPX genes
PAR genes
Expression sex bias
male
female
male
female
24
2
41
13
29
1
escape
inactive
Count
40
30
20
10
Female–biased
Female–biased
Male–biased
Enhancer sex-bias
